# Decentralized Multi-Robot Navigation Based on Deep Reinforcement Learning and Trajectory Optimization

**DOI:** 10.3390/biomimetics10060366

**Published:** 2025-06-04

**Authors:** Yifei Bi, Jianing Luo, Jiwei Zhu, Junxiu Liu, Wei Li

**Affiliations:** 1 College of Foreign Languages, University of Shanghai for Science and Technology, Shanghai 200093, China; yifeibi@usst.edu.cn; 2 College of Intelligent Robotics and Advanced Manufacturing, Fudan University, Shanghai 200433, China; 22210860108@m.fudan.edu.cn (J.L.); 21210860121@m.fudan.edu.cn (J.Z.); 3 Guangxi Key Laboratory of Brain-Inspired Computing and Intelligent Chips, Guangxi Normal University, Guilin 541001, China; liujunxiu@gxnu.edu.cn

**Keywords:** multi-robot navigation, reinforcement learning, graph neural network, obstacle avoidance, nonlinear optimization

## Abstract

Multi-robot systems are significant in decision-making capabilities and applications, but avoiding collisions during movement remains a critical challenge. Existing decentralized obstacle avoidance strategies, while low in computational cost, often fail to ensure safety effectively. To address this issue, this paper leverages graph neural networks (GNNs) and deep reinforcement learning (DRL) to aggregate high-dimensional features as inputs for reinforcement learning (RL) to generate paths. Additionally, it introduces safety constraints through an artificial potential field (APF) to optimize these trajectories. Additionally, a constrained nonlinear optimization method further refines the APF-adjusted paths, resulting in the development of the GNN-RL-APF-Lagrangian algorithm. By combining APF and nonlinear optimization techniques, experimental results demonstrate that this method significantly enhances the safety and obstacle avoidance capabilities of multi-robot systems in complex environments. The proposed GNN-RL-APF-Lagrangian algorithm achieves a 96.43% success rate in sparse obstacle environments and 89.77% in dense obstacle scenarios, representing improvements of 59% and 60%, respectively, over baseline GNN-RL approaches. The method maintains scalability up to 30 robots while preserving distributed execution properties.

## 1. Introduction

The collaboration of a multi-robot system has played an important role in enhancing the autonomy of robots’ decision-making capabilities, and it has found wide applications in areas such as exploration [1,2,3], surveillance [4], search and rescue [5], transportation [6], and agriculture [7,8]. Compared to a single robot, a multi-robot system offers better spatial distribution and can achieve more comprehensive system performance.

Multi-robot navigation draws significant inspiration from collective behaviors observed in biological systems. Flocking birds, schooling fish, and swarming insects demonstrate remarkable abilities to navigate complex environments while avoiding collisions through simple local interaction rules. These biological systems exhibit emergent global behaviors arising from local sensing and decision making, which directly motivates our distributed approach.

Our framework incorporates several biomimetic principles: (1) local information aggregation—similar to how birds in a flock only observe nearby neighbors, our GNN-based approach aggregates information from local robot neighborhoods; (2) repulsive force mechanisms—analogous to the collision avoidance behaviors in animal swarms, our APF component generates repulsive forces to maintain safe distances; and (3) adaptive path planning—mimicking how animals adjust their trajectories based on environmental feedback, our nonlinear optimization continuously refines robot paths. This bio-inspired approach enables robust, scalable navigation without requiring centralized coordination, closely paralleling the distributed nature of biological collective motion.

In multi-robot systems, ensuring that robots do not collide with each other or with obstacles is a critical issue. Robots need to move from their starting positions to given targets, avoiding collisions to ensure safety. Currently, there are two main approaches to solving this problem: centralized methods and distributed methods. Table 1 summarizes the key characteristics of these approaches.

In centralized methods, it is generally assumed that there is a centralized controller that gathers information about the states of all robots (such as positions, velocities, and targets) and the environment [9,10]. Using this information, the controller creates a global plan and calculates the movements for each robot to avoid obstacles. While this method is relatively reliable in terms of safety, it requires more computation time and resources to plan optimal actions for all robots as the number of robots increases, which poses a significant burden for algorithm deployment.

For large robot groups, distributed multi-robot obstacle avoidance strategies are often more effective due to their lower computational costs and greater applicability [11,12,13,14]. In distributed methods, each robot makes independent decisions while considering a small amount of information from other robots as a reference for its own movement. This approach is highly suitable for large robot groups.

**Figure 1:**
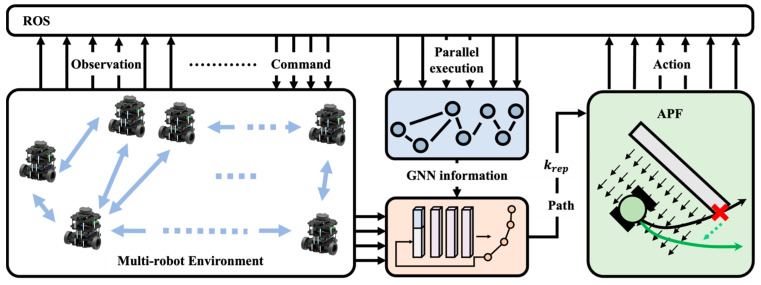
The framework of the multi-robot navigation system. It adopts a centralized training and decentralized execution scheme. A graph neural network is used to aggregate the high-dimensional features fed into the RL policy for generating a path. The APF and nonlinear optimization are used for trajectory refinement to enhance safety.

In light of the advantages of distributed obstacle avoidance strategies, this paper focuses on distributed multi-robot obstacle avoidance strategies. A graph neural network is used to aggregate the high-dimensional features of the multi-robot system, which are then input into a deep reinforcement learning policy network to generate paths. However, relying solely on neural networks for obstacle avoidance cannot guarantee the safety of the robots. To enhance robot safety and reduce the number of collisions, this paper draws inspiration from the artificial potential field (APF) algorithm. It leverages the repulsive forces derived from surrounding obstacles to optimize the reference trajectory output by the deep reinforcement learning model and defines a constrained nonlinear optimization problem, transforming this adjustment strategy into an optimization solution. This paper refers to this approach as the GNN-RL-APF-Lagrangian algorithm. Finally, the performance and behavior of the aforementioned algorithm are tested in a series of complex environments, thus verifying the feasibility of integrating an APF and nonlinear optimization into deep reinforcement learning for path generation. The proposed framework for multi-robot navigation is shown in Figure 1.

The main contributions of our work include the following:

- A novel GNN-RL-based decentralized multi-robot navigation framework using path generation is proposed, which is trained with deep reinforcement learning.
- An APF and nonlinear optimization are integrated into the optimization of the generated trajectory to ensure the safety of the multi-robot system.
- The scalability experiment of the multi-robot system and the obstacle avoidance experiment of a random obstacle scene are carried out to verify the effectiveness of the proposed method.

## 2. Related Work

In recent years, reinforcement learning (RL) has emerged as a promising approach to address the challenges of multi-robot navigation [15,16]. It has demonstrated significant potential in achieving navigation in complex and unstructured environments, as it can handle complex decision-making problems with a high-dimensional state and action spaces.

Chen et al. [17] proposed the CADRL algorithm for the decentralized multi-agent collision avoidance where a value network is deployed to output the action given the agents’ observable and hidden states. The robot can generate a path to its goal by repeatedly maximizing a one-step lookahead value based on the supervised training value network. In the study of [18], they consider navigation in pedestrian-rich environments and propose the SA-CADRL algorithm in which the social norms are introduced into the reward function for the value network training. Han et al. [19] propose a distributed approach for multi-robot navigation combining the reciprocal velocity obstacle (RVO) and DRL. Reciprocal collision avoidance is encouraged by incorporating the concept of RVO into the state space and reward functions. Cui et al. [20] also consider the safety of the multi-robot team. A control barrier function (CBF) is designed to optimize the action commands from the policy network.

Thumiger et al. [21] use LSTM and a gradient-inspired reward function to solve the decentralized collision avoidance problem. Based on the PPO algorithm and the paradigm of centralized training and decentralized execution, Wang et al. [22] proposed a fully distributed collision detection and avoidance method for multi-UAV. By incorporating the Cramér–Rao lower bound (CRLB) of the joint measurement likelihood function, Moon et al. [23] quantify the optimal UAV control actions by a reward function considering both the CRLB of the entire system and each UAV’s individual contribution to the system, thus improving the tracking accuracy by ensuring the reception of high-quality LoS measurements with high probability. Inspired by flocks of starlings (*Sturnus vulgaris*), Ourari et al. [24] propose a new, scalable observation model following a biomimetic nearest-neighbor information constraint that leads to fast learning and good collision avoidance behavior, and integrate collision avoidance with arbitrary tasks such as package collection and formation change.

## 3. Problem Statement

### 3.1. Trajectory Representation

**Figure 2:**
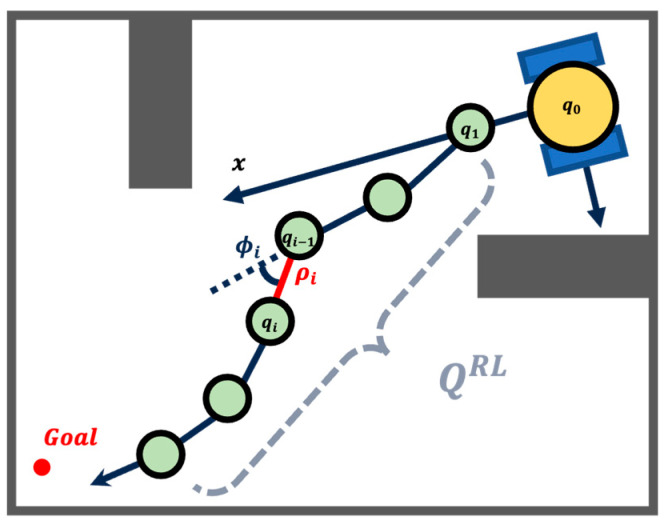
The representation of path points.

For each robot, we use a set of points $Q≔\left\{q_{1},\ldots ,q_{n}\right\}$ to represent the generated path. The $i^{th}$ point $q_{i}\left(\rho_{i},\phi_{i}\right)$ of the generated path is represented as the polar coordinate expression in the local coordinate system of the robot, where $\rho_{i}$ and $\phi_{i}$ are the radial coordinate and the angular coordinate with respect to the previous point $q_{i-1}\left(\rho_{i-1},\phi_{i-1}\right)$, respectively. For the first point $q_{1}$, its coordinates are generated relative to the origin of the robot’s local coordinate system. Figure 2 illustrates the relative relationship among the coordinates of the generated path points. In a multi-robot navigation scenario, the trajectory of a single robot is optimized by the RL policy, which will be further discussed in the following section.

### 3.2. Multi-Robot Weighted Adjacency Matrix

A group of N robots is modeled by an undirected graph $G=\left(V,E\right)$, where $V≔\;\left\{1\ldots N\right\}$ denotes the set of $\left|V\right|=N$ vertices, and E⊂V×V denotes the set of edges. In graph G, vertex i represents robot i whose position vector is $p_{i}=\left[x_{i},y_{i}\right]\in R^{2}$ and edge $e_{ij}\in E$ means the communication between vertex i∈V and vertex j∈V. The graph G is associated with an adjacency matrix $A={\left\{a_{ij}\right\}}_{N\times N}$ whose element $a_{ij}$ is the weight of edge $e_{ij}$. This weight is given by the following:

(1) $$a_{ij}=||p_{i}-p_{j}{\left|\right|}_{2},\;e_{ij}=\left(i,j\right)\in E$$

where ‖·‖_2_ denotes the Euclidean norm.

For multi-robot obstacle avoidance, this paper uses graph neural networks to aggregate neighborhood information and aggregate the high-dimensional perception features extracted by each robot. At the same time, since the edge features of the graph also contain a lot of important information, we define the weighted adjacency matrix $A_{c}={\left\{c_{ij}\right\}}_{N\times N}$, and use the edge information as the feature coefficient $c_{ij}$ to measure the relative distance between robots, thereby taking edge features into account in information aggregation. For robot i and robot j, the coefficient $c_{ij}$ is defined as follows:

(2) $$c_{ij}=e^{-a_{ij}}$$

e is the base of the natural logarithm.

## 4. Approach

### 4.1. Multi-Robot Path Generation

The multi-robot path generation problem can be formulated as a Partial Observable Markov Decision Process (POMDP) [25]. It is formally defined by the tuple <S,O,A,P,T,r,γ>, where S is the state space, O is the observation space, A is the action space, $P\left(s^{\prime }|s,a\right):S\times A\times S\to \left[0,1\right]$ is the state transition that maps state s and action a to the new state $s^{\prime }$, $T\left(o|s^{\prime },a\right):S\times A\times O\to \left[0,1\right]$ is the observation probabilities conditioned on the reached state $s^{\prime }$ and taken action a, $r\left(S,A\right):S\times A\to R$ is the reward function, and $\gamma \in \left(0,1\right)$ is the discount factor. The objective of RL is to learn an optimal policy $\pi_{\theta }$ with parameters θ, which maximizes the expected cumulative discounted rewards $J\left(\theta \right)$,

(3) $$J\left(\theta \right)=E\left[\sum_{t=0}^{T}\gamma^{t}r\left(s_{t},a_{t}\right)\right]$$

where T is the horizon of an episode.

In this paper, we consider a group of N homogeneous robots with the same radius $R_{s}$ whose objective is to navigate to a target location while avoiding each other and obstacles. At each time step t, each robot obtains observation from onboard sensors and other robots to estimate its own state. In our case, the observation $o^{t}$ contains the 2D laser scan data $o_{d}$, the relative goal position $o_{g}$, and the positions of other robots $o_{p}$. With the observation $o^{t}$ given as input, the policy $\pi_{\theta }$ executed on each robot with shared parameters outputs the action $a^{t}$, i.e., the generated path $Q_{t}$, for the robot to follow.

(1) Observation Space: The observation $o_{t}$ for each robot consists of 3 parts: the latest 3 frames of the 2D laser scan data $o_{d}$, the relative goal position $o_{g}$ and the positions $o_{p}$ of other robots in group. The 2D laser scan data sampled 180 measurements from a $360^{\circ }$ LIDAR with the orientation of the robot as zero. The position information is Cartesian coordinate, converted from the world coordinate system to the local robot coordinate system.
(2) Action Space: At each time step, a path $Q_{t}$ was generated for the robot to follow. The action $a_{t}$ therefore is represented by the generated path $Q_{t}$, which consists of n points. All these points are represented as polar coordinates and were also transformed into the local robotic coordinate system as mentioned in Section 3.1.
(3) Reward Design: To enable multi-robot group to navigate to the target locations while maintaining the formation and avoiding the collision, we designed the reward function $r\left(o_{t},a_{t}\right)$ to estimate the generated path and the robot’s current state:
  (4) $$r=r_{c}+r_{g}+r_{s}$$

The first part $r_{c}$ was designed to penalize the collisions among robots and obstacles. Since the action of each robot is a generated path, it was required to judge whether the path conflicts with obstacles. We used $d_{path}$ to denote the minimal distance between the positions of obstacles scanned by LIDAR and each line segment linked by the generated points, $d_{robot}$, to denote the minimal distance between current robot and other robots, and $d_{obs}$ to denote the minimal distance between current position and obstacles. If one of the metrics exceeds the corresponding threshold, then it is regarded as a collision, that is

(5) $$r_{c}=\left\{\begin{array}{c} -30,if\;d_{path}\;or\;d_{obs}<R_{s},\;or\;d_{robot}<2R_{s}\\ 0,\;otherwise \end{array}\right.$$

where $R_{s}$ is the radius of each robot. To encourage the robot to reach the goal position, we calculate the distance between the generated path points and the goal position as part of the reward function:

(6) $$r_{g}=\sum_{i=0}^{n}\frac{d_{goal}-d_{i}}{i}$$

where $d_{goal}$ is the distance between current position and goal position, and $d_{i}$ represents the distance between $d_{i}$ point and goal position.

The smoothness of the generated path was another consideration in our task. To limit excessive variation in the angles of the generated path points, we adopted the sum of the squares of the angular coordinates of all points on the entire path as the reward function $r_{s}$:

(7) $$r_{s}=-w_{s}\sum_{i=1}^{n}\phi_{i}^{2}$$

where $w_{s}$ is a tuning parameter, and $\phi_{i}$ is the angle coordinate of $i^{th}$ point $q_{i}\left(\rho_{i},\phi_{i}\right)$ in the generated path Q. And we empirically set $w_{s}=5\times 10^{-4}$.

Following a centralized training and decentralized implementation scheme, the shared policy $\pi_{\theta }$ can be derived from a number of DRL algorithms originally used for each single robot and can be implemented independently on each robot as the observations are only from local information.

### 4.2. Learning Local Information Based on GCN

Graph neural networks are deep learning methods based on graph data structures. They can be used to handle various tasks on graphs. In the field of robotics, graph neural networks are often used to aggregate various features of robots for multi-robot tasks. Graph convolutional neural networks (GCNs) can learn the topological features of graphs by performing convolution operations in graph structures, utilizing the connections between nodes, and considering node neighborhood information during feature propagation. GCN can fuse the local neighborhood information of nodes with the global information of the entire graph. This information fusion mechanism enables GCNs to have a more comprehensive understanding of the state of the entire system when dealing with complex relationships between multiple robots, so that the network is not limited to the individual information of each robot and has great advantages in applications in multi-robot scenarios.

We use GCNs to aggregate the feature information of robots to enrich the input and improve the performance of the algorithm. $H_{l}$ is defined as the input of the $l_{th}$ layer of the neural network. The function $g\left(H^{l},D,W^{l}\right)$ represents the GCN layer, and the specific form of the features of the ${\left(l+1\right)}_{th}$ layer is

(8) $$H^{l+1}=g\left(H^{l},D,W^{l}\right)=\sigma \left(D^{-\frac{1}{2}}A_{c}D^{-\frac{1}{2}}H^{l}W^{l}\right)$$

where $W^{l}$ is the parameter of the $l_{th}$ layer of the neural network, and $\sigma \left(\cdot \right)$ is the activation function. The matrix D is the weighted degree matrix of the graph, which is a diagonal matrix satisfying $D=\{d_{ii}{\}}_{N\times N}$, whose element $d_{ii}=\sum_{j}\{c_{ij}\}$ is the sum of each row of the weight adjacency matrix. Normalizing the weight adjacency matrix $A_{c}$ (as mentioned in Section 3.2), using the diagonal matrix, helps to unify the scale of each robot feature vector. The above mathematical expression of the graph convolutional network is based on the graph Fourier transform theory and is simplified by using the first-order Chebyshev polynomial approximation to prevent the gradient from disappearing or exploding.

### 4.3. Trajectory Optimization Based on APF

Artificial potential field (APF) is a method commonly used for path planning and obstacle avoidance. This method simulates the movement of an object in a potential field, and, by introducing a virtual potential field in the environment, the object is affected by a force and moves in the potential field. In the field of robotics, APF is mainly used for path planning and obstacle avoidance, enabling robots to navigate autonomously in complex environments.

The basic idea of the artificial potential field method is to define a potential field so that the robot is affected by a virtual potential field when moving in the environment. The potential field usually includes two components: attractive potential and repulsive potential. The attractive potential is often used to guide the robot towards the target, while the repulsive potential is used for the robot to avoid obstacles. This paper mainly uses the integration of repulsive potential into the trajectory optimization part of multi-robot obstacle avoidance.

For a single robot in a robot group, its position coordinates are represented by $p=\left[x,y\right]$. During the movement process, the robot will obtain the coordinates of the obstacle through the single-line laser radar it carries. For each obstacle $O_{i}$, the distance between the robot and the obstacle is $d_{i}=||p-O_{i}|{|}_{2}$. Based on the distance information obtained, the repulsive potential energy $U_{rep}$ is defined to satisfy the following:

(9) $$U_{rep}\left(O_{i}\right)=\left\{\begin{array}{c} \frac{1}{2}k_{rep}{\left(\frac{1}{d_{i}}-\frac{1}{R_{safe}}\right)}^{2},\;d_{i}\;\leq {\;R}_{safe}\\ 0,\;d_{i}>R_{safe} \end{array}\right.$$

where $k_{rep}$ is the repulsion coefficient, $R_{safe}$ is the safety radius, and $U_{rep}\left(O_{i}\right)$ will be greater than 0 only when the distance between the robot and the obstacle is less than the safety radius. After calculating the repulsive potential energy, the repulsion $f_{rep}$ in each coordinate axis direction can be obtained by taking the gradient of $U_{rep}\left(O_{i}\right)$ with respect to the robot’s current position p:

(10) $$f_{rep}\left(O_{i}\right)=\left\{\begin{array}{c} -k_{rep}\left(\frac{1}{d_{i}}-\frac{1}{R_{safe}}\right)\frac{1}{d_{i}^{2}}\frac{{\partial d}_{i}}{\partial p},{\;d}_{i}\leq {\;R}_{safe}\\ 0,\;d_{i}>R_{safe} \end{array}\right.$$

It is obvious that the magnitude of each component of the repulsive force can be adjusted by adjusting the repulsive force coefficient $k_{rep}$, thereby adjusting the magnitude of optimization. The reinforcement learning generated path optimization integrating artificial potential field uses the reinforcement learning output repulsive force coefficient $k_{rep}$ to dynamically adjust the repulsive force magnitude according to environmental information, thereby outputting the corresponding optimized path for obstacles as shown in Figure 1.

### 4.4. Neural Network Structure

**Figure 3:**
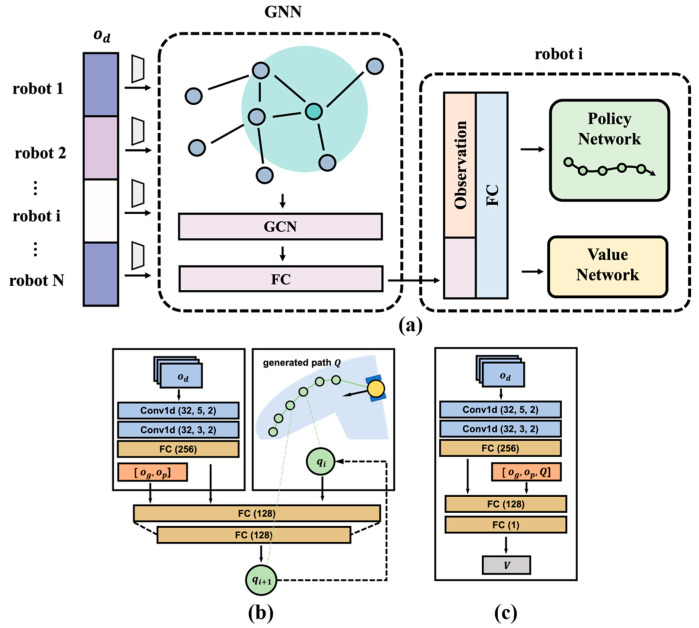
(**a**) The network structure of multi-robot path generation using GNN. Conv1d represents the 1D convolution layer, and FC represents the fully connected layer. (**b**) Policy network, which generates the n points path. The inputs are the latest 3 frames of raw laser data $o_{d}$, the positions of goal $o_{g}$ and other robots $o_{p}$. (**c**) Value network. The inputs are $o_{d},o_{g},o_{p}$, and the generated path is Q.

We used an actor–critic network to iteratively generate path points, which is similar to our previous work [26]. Figure 3 represents the structure of the policy network. In each iteration, the 2D laser scan data $o_{d}$ were first processed by two 1D convolution layers (Conv1d) and one fully connected layer (FC). Then, it was concatenated with other observation data $o_{g}$, $o_{p}$, and the path point $q_{i-1}$ from the previous iteration, which were fed into one hidden FC layer with leaky rectified linear units (ReLUs) as activation functions (for the first iteration, the path point $q_{0}$ was set as an empty vector). The output of the network contains the mean and standard deviation following a Gaussian distribution. The robot randomly generates path according to this distribution for online exploration.

The value network adopted a Conv1d to extract laser data and connected this feature vector with other observations to obtain value estimates through a hidden and an output FC layer.

### 4.5. Path Adjustment Based on Nonlinear Optimization

Although the GNN-RL-APF algorithm optimizes the trajectory output by reinforcement learning through an artificial potential field, it is still possible to generate unsafe trajectories because its step size parameters are still inconsistent in different situations. In order to further improve the safety of the GNN-RL-APF algorithm, we perform path optimization based on the path it outputs. The optimization process involves adjusting the positions of the generated path points within a certain range to meet the constraints and output a safer trajectory. Specifically, for the path $\pi_{\theta }$, after reinforcement learning and APF output, the optimized path is defined as $Q_{opt}$. The goal of optimization is to adjust the path as little as possible while ensuring safety. This goal can be formally expressed as

(11) $$\underset{Q_{opt}}{min}||Q_{opt}-\pi_{\theta }{\left|\right|}_{2}^{2},\;s.\;t.\;{\;f}_{c}\left(o_{d},Q_{opt}\right)\geq 0)$$

The function $f_{c}\left(o_{d},Q_{opt}\right)$ is the defined cost function, which is related to the geometric constraints of the path points and mainly measures the collision between the path and obstacles. It is defined as follows:

(12) $$f_{c}=\underset{\mathrm{path}}{\max}|{|d}_{path}-d_{safe}{\left|\right|}_{2}^{2},\;if\;d_{path}<d_{safe}$$

where $d_{path}$ is the distance between the path segment and the discretized obstacle point, and $d_{safe}$ is the pre-defined safety distance.

Since geometric constraints are often non-convex, the problem is difficult to solve. To overcome this challenge, we convert the problem into a Lagrangian dual problem [27] to facilitate the subsequent solution process. First, the Lagrangian function is defined as follows:

(13) $$L\left(Q_{opt},\pi_{\theta },\lambda \right)=||Q_{opt}-\pi_{\theta }{\left|\right|}_{2}^{2}-\lambda f_{c}\left(o_{d},Q_{opt}\right)$$

where λ is the Lagrange multiplie. The constrained optimization problem (Equation (11)) can be converted into the following equivalent form:

(14) $$\underset{Q_{\mathrm{opt}}}{\min}\underset{\lambda \geq 0}{\max}L\left(Q_{opt},\pi_{\theta },\lambda \right)$$

Then, we get the unconstrained Lagrangian dual problem:

(15) $$\underset{\lambda \geq 0}{\max}\underset{Q_{\mathrm{opt}}}{\min}L\left(Q_{opt},\pi_{\theta },\lambda \right)$$

In the solution, $L\left(Q_{opt},\pi_{\theta },\lambda \right)$ is first minimized (by iteratively solving the gradient minimization objective function until the gradient is 0 or the number of iterations is reached), and then λ is maximized (also using iterative solution), and finally the optimized path $Q_{opt}$ is obtained.

**Figure 4:**
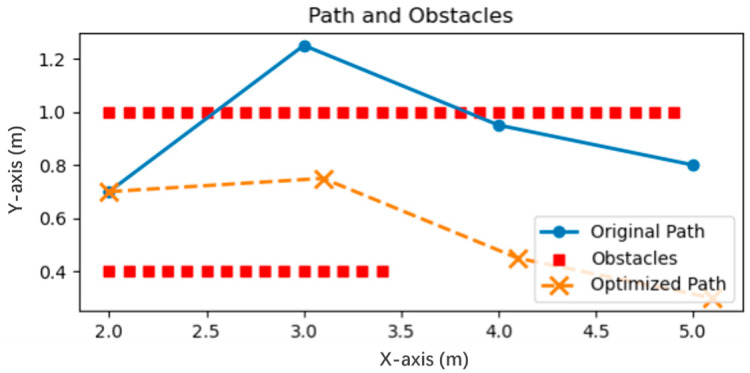
Comparison before and after path optimization.

Figure 4 shows the path comparison before and after path optimization in simulation. The red discrete points represent obstacles, the blue line segments are the assumed planned reference paths, and crossing obstacles at this time represents an unsafe state, while the orange dotted line is the optimized path obtained by solving (Equation (15)), the Lagrangian dual problem.

It can be observed that the optimized and adjusted path points avoid obstacles and find a feasible path while maintaining safety. At the same time, the path adjustment based on nonlinear optimization is completely distributed, and each robot only uses its own local perception information and planning data to generate a safe trajectory. This path optimization method not only allows the robot to avoid potential collision risks but also maintains a certain safety distance, improves the safety and robustness of navigation, and its local computing characteristics also make the algorithm’s scalability unrestricted, providing a more reliable path planning strategy for the robot’s navigation in complex scenes.

### 4.6. Policy Training

**Figure 5:**
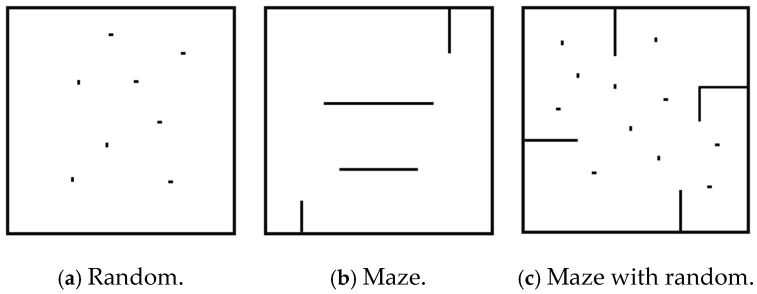
Three types of training maps.

We deployed our algorithm in the simulation environment for policy training where sufficient samples are available. To avoid overfitting, we randomly generated 3 types of maps as the training environment, as shown in Figure 5. In each map, the location and number of obstacles were changed randomly. For the policy update, we use the Multi-process Proximal Policy Optimization (PPO) [28] to find the optimal policy.

## 5. Experiment

### 5.1. Evaluation Metrics

To evaluate the performance of those methods, the following four metrics are used for further analysis.

- Success Rate: The rate of the robot team to reach the goal position without collision. If a collision occurs, the current test is immediately terminated and marked as a failure.
- Average Trajectory Length: The average length of the trajectory of the robot in the team that successfully moves to the target.
- Average Time Step: The average time steps for the robot to travel to the goal.
- Average Time Cost: The average time cost for the robot to travel to the goal.

### 5.2. Multi-Robot Mutual Obstacle Avoidance Algorithm Comparative Experiment

In this section, a comparative experiment was conducted to evaluate the performance of the GNN-RL and GNN-RL-APF algorithms in handling mutual obstacle avoidance among multiple robots on an obstacle-free map. The robot we use is Turtlebot3, equipped with RP-A2 single-line LiDAR. The robot is controlled to move by giving it linear speed and angular speed instructions. The experiment used a 15 m × 15 m open map without obstacles. The robots were designed to perform a collision avoidance task by moving towards the center of the map simultaneously, avoiding each other, and ultimately reaching target positions that are the exact opposites of their starting coordinates.

The purpose of this design was to simulate a complex multi-robot obstacle avoidance scenario to maximize the testing of the algorithms’ performance. Since the robots start from the diagonals and converge at the center of the map, there is a high likelihood of collisions occurring among the robots. In this scenario, the obstacle avoidance algorithms need to possess strong dynamic handling capabilities. Additionally, as the number of robots increases, the complexity of the experiment significantly rises. By conducting this experiment, the algorithms can be comprehensively evaluated in terms of their performance in multi-robot collaborative obstacle avoidance tasks, including their adaptability to dynamic environments, ability to handle collision situations, and scalability as the number of robots increases.

The experimental results are shown in Table 2, where the performance of each algorithm was tested in mutual obstacle avoidance tasks with 2, 3, 4, 6, and 8 robots. The evaluation metrics included the success rate, average trajectory length, average time steps, and average time consumption (the success rate column includes the collision rate in parentheses; if their sum is not 100%, it indicates that some robots experienced a timeout).

In all test scenarios, GNN-RL-APF demonstrated superior performance, achieving a 100% success rate consistently. Additionally, GNN-RL-APF significantly reduced the time consumed for the same tasks compared to the GNN-RL algorithm, indicating its excellent dynamic handling and efficient decision-making capabilities in complex situations.

In contrast, the GNN-RL algorithm only achieved a 100% success rate in the scenario with four robots. Its performance declined with different numbers of robots. Notably, in the three-robot and six-robot environments, the GNN-RL algorithm experienced timeouts, primarily because it struggled to make stable decisions in dynamic environments, causing robots to deviate too far from their target positions and ultimately resulting in task timeouts. This also led to increased average path lengths and time consumption, indicating relatively poorer performance.

Further analysis reveals that the superiority of GNN-RL-APF in multi-robot cooperative obstacle avoidance tasks is mainly due to the incorporation of the APF mechanism. An APF allows robots to maintain a certain level of coordination while avoiding obstacles, effectively preventing collisions and chaos. In contrast, the GNN-RL algorithm appears less capable of handling complex dynamic environments, leading to performance degradation and timeouts. This highlights the superiority of GNN-RL-APF in multi-robot cooperative tasks, especially in scenarios with certain complexity and dynamic nature, where it shows remarkable adaptability.

**Figure 6:**
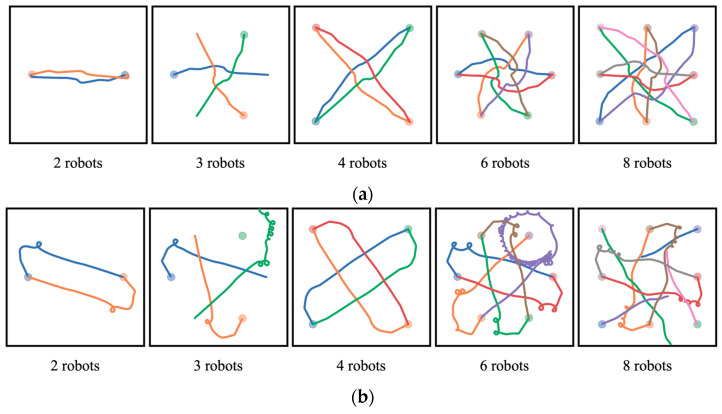
Movement trajectory diagrams of robot groups with different quantities. (**a**) Movement trajectory diagrams of robot groups with different quantities using the GNN-RL-APF algorithm. (**b**) Movement trajectory diagrams of robot groups with different quantities using the GNN-RL algorithm.

Figure 6 shows the movement trajectory diagrams of robots using the GNN-RL and GNN-RL-APF algorithms in the stage environment. The circular shaded areas corresponding to the trajectory colors represent the target positions. As indicated by the trajectories in the figure, the GNN-RL-APF algorithm performs excellently in completing the task. When the robots converge at the central position, despite the environment becoming extremely complex and dynamically changing, the GNN-RL-APF algorithm dynamically adjusts the generated paths based on the obtained repulsive forces. Thus, the jittering of trajectories occurs at the center of the map due to the mutual obstacle avoidance among robots. However, when robots move away from the central dense area, the GNN-RL-APF algorithm generates more stable trajectories based on the positions of the target area and the boundary information obtained from radar scans. Therefore, compared to the trajectories in the central area, the trajectories become smoother when approaching the target position.

This demonstrates that the GNN-RL-APF algorithm exhibits good dynamic performance, generating safe and collision-free trajectories based on current observation information and optimized parameters of APF while also flexibly adjusting to maintain a certain level of smoothness in dynamic environments.

In contrast, the performance of the GNN-RL algorithm shows significant differences across environments with different numbers of robots. It only presents safe and smooth trajectory states when the environment contains four robots. In other environments, there are instances where robots rotate in place and make erratic decisions, leading to a decrease in trajectory quality or even collisions with the boundaries. This is because the training environment used for the GNN-RL algorithm consisted of four robots, allowing it to learn relevant features specific to this environment. However, as the environment changes and the number of robots varies, the performance of the algorithm sharply declines, indicating weak scalability and transferability of the GNN-RL algorithm. In contrast, the GNN-RL-APF algorithm can adapt to various numbers of multi-robot environments and maintain excellent decision-making capabilities, ultimately outputting high-quality paths for robots to complete the designated tasks.

### 5.3. Multi-Robot Large-Scale Mutual Obstacle Avoidance Experiment

To test the scalability of the GNN-RL-APF algorithm, this section continues testing in environments with groups of 10, 20, and 30 robots. The map size is set to 20 m × 20 m, and both the initial positions and target positions of the robots are randomly generated. The test results are shown in Table 3.

According to the data in Table 3, the GNN-RL-APF algorithm maintains excellent performance with a success rate as high as 95.38% in the environment with 10 robots. As the number of robots increases, the success rate gradually decreases due to the increased density of the map. However, even with 30 robots, the algorithm still manages to enable most robots to successfully reach their target positions. Additionally, as the number of robots increases, the algorithm’s time consumption also increases accordingly, as individual robots need to consider more decision factors and are influenced by communication.

**Figure 7:**
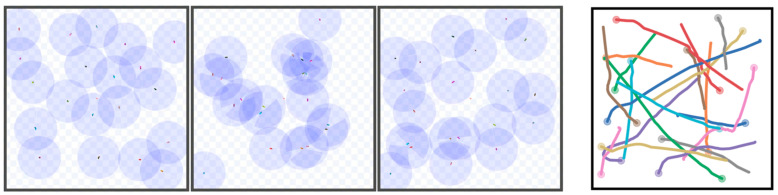
Operational diagrams of 20 robots navigating mutual obstacle avoidance from random positions.

Figure 7 also demonstrates the operational and trajectory diagrams of each robot in the environment with 20 robots. It shows that, even in densely packed environments, the robots can maintain a high success rate in decision making and reach their target points. This experiment confirms that GNN-RL-APF possesses robust scalability, maintaining superior performance even in large-scale robot groups.

### 5.4. Comparative Experiments on Multi-Robot Mutual Obstacle Avoidance in Complex Environments

Although the GNN-RL-APF algorithm has shown outstanding performance in multi-robot mutual obstacle avoidance scenarios on obstacle-free maps, it is necessary to evaluate the algorithm’s performance in complex obstacle environments to comprehensively assess its capability in multi-robot mutual obstacle avoidance scenarios on maps with random obstacles.

In this section, a series of randomly generated obstacle maps was used to test the robot obstacle avoidance capabilities in complex obstacle environments. In addition to testing the GNN-RL and GNN-RL-APF algorithms, the GNN-RL-APF-Lagrangian algorithm proposed in Section 4.5 was also tested. This algorithm further adjusts the output paths based on the GNN-RL-APF algorithm, using the theory of nonlinear optimization to solve the problem and output new trajectories, focusing on improving the safety of paths. The results of the test experiments on sparse obstacle maps are shown in Table 4.

According to the results in Table 4, the GNN-RL-APF-Lagrangian algorithm achieved a success rate of 96.43%, higher than that of the GNN-RL-APF algorithm, while the performance of the GNN-RL algorithm was the lowest, with only 37.5%. These results clearly demonstrate the performance improvement of the proposed path adjustment method based on nonlinear optimization in extreme environments. The high success rate of the GNN-RL-APF-Lagrangian algorithm indicates that, in scenarios where static obstacles coexist with dynamic robots, the algorithm can generate paths that are safer and more reliable.

In terms of path length, the paths generated by GNN-RL-APF and GNN-RL-APF-Lagrangian are relatively close, with GNN-RL-APF-Lagrangian slightly longer than those of the GNN-RL-APF algorithm. This is because the addition of nonlinear optimization in the GNN-RL-APF-Lagrangian algorithm leads to path length increases in extreme situations, as it chooses detours to avoid obstacles. The path length of the GNN-RL algorithm is the shortest, mainly because its obstacle avoidance capability is weaker, resulting in successful navigation in simpler scenarios without the need for complex obstacle avoidance, thus reducing the chances of detours and resulting in shorter path lengths. It is worth noting that only the path lengths at successful arrivals are considered in this section.

In terms of time consumption, the GNN-RL-APF-Lagrangian algorithm generally requires more time to adjust paths due to the need to solve nonlinear optimization problems. Therefore, it exhibits the highest time consumption. However, it is important to note that, due to differences between simulation environments and real-world scenarios, robot movements in simulations may be accelerated, and the optimization time is measured based on real-world scenarios. Since the time scales of the two cannot be unified, there may be an overestimation of the time consumption of the GNN-RL-APF-Lagrangian algorithm. Therefore, the time consumption statistics are only used as references for algorithm comparison and cannot be used as actual data.

**Figure 8:**
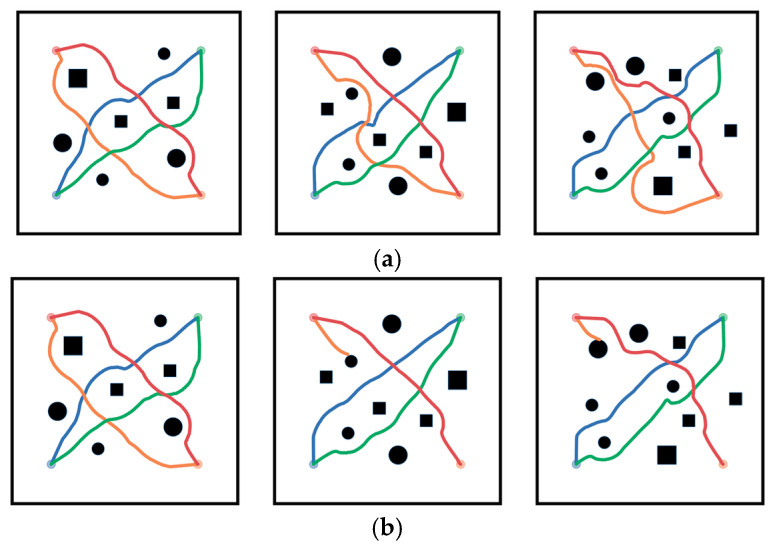
Trajectory diagrams of multi-robot groups in environments with random obstacles. (**a**) Movement trajectory diagrams of multi-robot groups in environments with random obstacles using the GNN-RL-APF-Lagrangian algorithm. (**b**) Movement trajectory diagrams of multi-robot groups in environments with random obstacles using the GNN-RL-APF algorithm.

Meanwhile, Figure 8 presents the trajectories of multi-robots moving in a random map environment. Four robots move towards each other, progressing diagonally. The circular areas with colors corresponding to the robot trajectories represent the target positions. In scenarios using the GNN-RL-APF-Lagrangian algorithm, when the distance between obstacles and robots is less than the safety distance, the algorithm adjusts and optimizes the current path so that the robots can cleverly navigate around and away from obstacles. The algorithm also considers both dynamic robots and static obstacles, balancing policy decisions and path optimization. As shown by the orange trajectory in the top right corner, significant detours occur. This is because the presence of nonlinear optimization causes the robots to change their original decision directions, and the robot regenerates paths based on this change.

In contrast, although the GNN-RL-APF algorithm generates repulsive forces and corresponding avoidance directions, there is a risk of getting stuck in local optima, potentially conflicting with paths generated by reinforcement learning. This can lead to failed obstacle avoidance or, even with avoidance tendencies, robots may collide due to constraints imposed by robot kinematics, as seen in Figure 8, where collisions occur due to delayed evasion.

To further test the performance of the proposed algorithm, we also conducted algorithm tests on a series of obstacle-dense test maps. The results are shown in Table 5. It can be observed that the success rates of all three algorithms have decreased, but the success rate of the GNN-RL-APF-Lagrangian algorithm still remains the highest, close to 90%. This is sufficient to demonstrate the excellent capability of the proposed GNN-RL-APF-Lagrangian algorithm in multi-robot obstacle avoidance in complex environments. However, the Lagrangian optimization component introduces computational overhead, particularly in dense obstacle environments. While maintaining O(*n*) complexity, the constant factor is higher than pure RL approaches. For scenarios requiring sub-10ms response times, the GNN-RL-APF variant (without Lagrangian optimization) may be preferable.

Overall, in dynamic environments, since all robots use the GNN-RL-APF algorithm, they generate repulsive forces when they meet, enabling flexible maneuvering. However, in environments where static obstacles coexist with dynamic robots, static obstacles do not move based on robot positions. This poses a risk of the GNN-RL-APF algorithm getting stuck in local optima, leading to a decline in its performance. Subsequently, the GNN-RL-APF-Lagrangian algorithm is aimed at addressing these issues by adding an extra layer of safety to the robot paths generated. Experimental results demonstrate a significant improvement in safety achieved. However, it is important to note that this performance improvement comes with some costs, including increased path lengths due to robot detours and increased computation time required for solving optimization problems. In practical applications, these factors need to be balanced when selecting an algorithm suitable for specific scenarios.

## 6. Conclusions

This paper investigated the multi-robot obstacle avoidance problem in complex dynamic scenarios using a framework combining GNN, RL, and trajectory optimization. By introducing artificial potential fields and nonlinear optimization to optimize paths generated by deep reinforcement learning, the proposed framework exhibited superior performance in various scenarios.

Firstly, the concept of artificial potential fields was introduced, along with the relevant formulas for repulsive potential energy and repulsive forces. Then, based on the repulsive force expression, a multi-robot algorithm framework was presented, using reinforcement learning to adjust the repulsive force coefficient to generate trajectories for multiple robots.

Subsequently, to address the issue of APF easily getting stuck in local optima, a nonlinear optimization problem was defined, with obstacle avoidance requirements as inequality constraints of the optimization problem. By utilizing optimization theory, the original optimization problem was transformed into a Lagrangian dual problem, simplifying the solution steps and speeding up the solution process.

Finally, a series of experimental tests were conducted on the proposed GNN-RL-APF and GNN-RL-APF-Lagrangian algorithms. In obstacle-free maps, the capabilities of the GNN-RL-APF algorithm in handling dynamic multi-robot obstacle avoidance simultaneously and its scalability to large robot groups were verified. The effectiveness of the GNN-RL-APF algorithm far exceeded that of the GNN-RL algorithm without APF optimization, maintaining a high success rate even in large-scale robot groups. In complex environments with random obstacles, the GNN-RL-APF-Lagrangian algorithm, with added safety measures, outperformed the GNN-RL-APF algorithm and alleviated the issue of APF getting stuck in local optima. However, the trade-off was the increased runtime consumption, which needs to be considered in practical applications.

In summary, the proposed GNN-RL-APF and GNN-RL-APF-Lagrangian algorithms demonstrated significant potential in handling multi-robot obstacle avoidance scenarios, not only in dynamic environments but also by adding safety guarantees for robots, and exhibited strong scalability. Additionally, this paper provided validation for extending the multi-robot framework based on GNN, RL, and trajectory optimization, offering insights into the development of a multi-robot coordination frame. In future work, the multi-robot navigation system will be explored in several key areas. In terms of hardware implementation, it is planned to deploy on heterogeneous robot teams consisting of ground vehicles and aerial drones for applications such as warehouse automation and search-and-rescue operations. To meet the sub-millisecond response requirements of time-critical applications, research on parallel computing architectures and GPU acceleration will be carried out to achieve real-time optimization. Regarding the enhancement of perception capabilities, the system will be extended to vision-based SLAM systems and integrated with semantic understanding functions to support navigation in human-populated environments. For more realistic deployment scenarios, efforts will be made to make the system adaptable to robot teams with varying capabilities, sizes, and kinematic constraints, improving the collaborative efficiency of heterogeneous teams. At the theoretical level, formal safety certificates and convergence guarantees will be developed to provide a solid theoretical foundation for the combined learning–optimization framework. The proposed framework represents a significant step towards practical, safe, and scalable multi-robot navigation systems, which have broad application prospects in autonomous logistics, environmental monitoring, collaborative robotics, and other fields.

## Figures and Tables

**Table 1:** Comparison of centralized vs. distributed multi-robot navigation approaches.

Aspect	Centralized Methods	Distributed Methods
Computational Complexity	O(*n^k^*) exponential growth	O(*n*) linear growth
Communication Requirements	High (global state sharing)	Low (local information only)
Scalability	Limited (<10 robots typically)	High (100 + robots possible)
Optimality	Global optimum achievable	Local optimum, good approximation
Robustness	Single point of failure	Fault-tolerant
Real-time Performance	Poor for large teams	High

**Table 2:** Comparative results of obstacle avoidance for obstacle-free map robots.

Number	Methods	Success Rate (%)	Average Trajectory Length (m)	Average Time Step	Average Time Cost (×10 s)
2 robots	GNN-RL	62.50	16.422	914	21.458
	GNN-RL-APF	100.00	10.264	583	14.281
3 robots	GNN-RL	71.67	16.639	915	22.982
	GNN-RL-APF	100.00	9.178	461	12.356
4 robots	GNN-RL	100.00	15.979	828	23.318
	GNN-RL-APF	100.00	13.534	658	19.009
6 robots	GNN-RL	85.00	16.213	802	27.292
	GNN-RL-APF	100.00	10.281	470	16.578
8 robots	GNN-RL	76.82	16.162	819	32.216
	GNN-RL-APF	100.00	12.087	555	22.072

**Table 3:** Results of mutual obstacle avoidance experiments among robots with random positions on obstacle-free maps.

Number	Methods	Success Rate (%)	Average Trajectory Length (m)	Average Time Step	Average Time Cost (×10 s)
10 robots	GNN-RL-APF	95.38	18.473	918	41.668
20 robots	GNN-RL-APF	86.67	17.441	646	75.842
30 robots	GNN-RL-APF	68.33	16.446	562	138.92

**Table 4:** Results of comparative experiments on multi-robot obstacle avoidance in sparse obstacle maps.

Methods	Success Rate (%)	Average Time Step	Average Trajectory Length (m)	Average Time Cost (×10 s)
GNN-RL	37.50	655	13.664	23.434
GNN-RL-APF	90.00	702	15.054	24.430
GNN-RL-APF-Lagrangian	96.43	709	15.308	100.795

**Table 5:** Results of multi-robot obstacle avoidance experiments in dense obstacle maps.

Methods	Success Rate (%)	Average Time Step	Average Trajectory Length (m)	Average Time Cost (×10 s)
GNN-RL	29.54	977	20.680	38.792
GNN-RL-APF	84.09	1077	20.849	30.068
GNN-RL-APF-Lagrangian	89.77	1075	20.873	104.578

## Data Availability

The original contributions presented in the study are included in the article and further inquiries can be directed to the corresponding authors.
